# The Relationship between Salivary Beta-2 Microglobulin and Uremia Intensity in Men with Chronic Renal Failure

**Published:** 2013-02-20

**Authors:** Mohammad Vahedi, Hossein Malekzadeh, Habib Haybar, Ali Reza Soltanian, Shermin Abdollahzadeh, Hojjat Yoosefi, Masoud Seyedian, Leila Yazdanpanah, Abrotan Saeid, Maryam Shabanpour Fooladi, Marziyeh Ghasemi

**Affiliations:** 1. Department of Oral Medicine, Dental Research Center, School of Dentistry, Hamadan University of Medical Sciences, Hamadan, Iran; 2. Department of Oral Medicine, School of Dentistry, Ahvaz Jundishapour University of Medical Sciences, Ahvaz, Iran; 3. Cardiovascular Research Center, Golestan Hospital, Ahvaz Jundishapour University of Medical Sciences, Ahvaz, Iran; 4. Department of Biostatistics and Epidemiology, School of Health, Hamadan University of Medical Sciences, Hamadan, Iran; 5. Department of Periodontics, School of Dentistry, Ahvaz Jundishapour University of Medical Sciences, Ahvaz, Iran; 6. Department of Internal Medicine, Ahvaz Jundishapour University of Medical Sciences, Ahvaz, Iran

**Keywords:** Chronic Renal Failure, Beta 2-Microglobulin, Uremia, Saliva

## Abstract

**Objective:**

This study defines the relationship between salivary beta-2 microglobulin (β2-M) and intensity of uremia in male patients diagnosed with chronic renal failure (CRF).

**Materials and Methods:**

In total of 42 males were enrolled in a case-control study. There were 21 cases of CRF and 21 control cases. We collected 10cc of saliva plus 5 cc of blood from all patients to determine β2-M, blood urea nitrogen (BUN) and creatinine (Cr) levels.

**Results:**

There was a correlation between the level of serum BUN and salivary urea in controls and patients, which was statistically significant for controls (p=0.028).The correlation between serum and salivary Cr was 0.195 in controls (p=0.398) and 0.598 in patients (p=0.006), which was statistically significant in patients. The correlation between serum and saliva was 0.133 (p=0.566) in controls and 0.078 (p=0.737) in patients, which was not statistically significant. The correlation between serum BUN and β2-M was 0.168 (p=0.469) in the control group and 0.629 (p=0.002) in patients, which was statistically significant in patients. The correlation between serum Cr and β2-M was 0.110 (p=0.635) in the control group and 0.678 (p=0.001) in patients, which was statistically significant in patients. The correlation between serum BUN and salivary β2-M was 0.093 (p=0.0690) in controls and 0.152 (p=0.152) in patients, which was not statistically significant. The correlation between serum Cr and salivary β2-M was 0.072 (p=0.070) in the control group and 0.286 (p=0.209) in patients, which was not statistically significant in either group.

**Conclusion:**

The results of the study indicated that salivary β2-M cannot be used as a noninvasive indicator to detect the severity of renal failure.

## Introduction

Kidneys excrete approximately 1.5 to 2.5 l of urine per day. Although the removal of toxic and waste products from the blood remains their major role, the kidneys are also essential for the production of hormones such as vitamin D, erythropoietin, and for the modulation of salt and water. Normal renal function can be maintained until approximately 50% of the nephrons per kidney are destroyed (1). Chronic renal failure (CRF) is the end result of a wide array of pathologic processes that reduce the total number of functioning nephrons to the point where dialysis or transplantation is necessary for survival. Uremic syndrome refers to the final stages of progressive renal insufficiency and results from functional derangements of many organ systems, although the prominence of specific symptoms may vary from patient to patient. Glomerulonephritis has been considered to be a leading cause of CRF, but due to progress in the prevention and treatment of this disease in recent years, diabetic nephropathy and hypertensive nephropathy are now the main causes (2). Two groups of symptoms are present in patients with uremic syndrome: symptoms referable to deranged renal excretory and regulatory function (fluid volume, electrolyte abnormalities, acid-base imbalance, retention of nitrogenous waste, and anemia) and a group of clinical symptoms that affect the cardiovascular, neuromuscular, gastrointestinal, immunologic, hematologic, metabolic, and dermatologic systems (1). The most common symptoms of renal failure include changes in urinary albumin- to-creatinine (Cr) ratio and an increase in the glomerular filtration rate, which leads to oral manifestation and disorders related to impaired renal function. The eruption of teeth may be delayed in children with renal failure (3, 4). In a study conducted in Brazil, 83% of patients onhemodialysis, peritoneal dialysis, and renal transplantation had oral disease and 87% had dental plaque (5).

Beta-2 microglobulin (β2-M) is a toxic substance found in the serum and saliva of patients with CRF. Accumulation of this protein may lead to amyloidosis, which is characterized by the progressive deterioration of joints resulting from deposits of amyloid fibers in joint areas as well as skeletal-muscle tissues (2-6). The presence of amyloidosis is seen in patients with 6-10 year history of dialysis (7). β2-M shows an increase in parallel with the development of CRF, leading to a decrease renal disease, the serum level of β2-M is between 20-50 mg/l (8). The normal level of this protein in the serum of healthy people is within 0-2.4 mg/l, and in the saliva of healthy people it is within 0-0.38 mg/l (9). Elevations in β2-M may be seen in disorders such as multiple myeloma, Sjӧgren syndrome, and some cancers (10). β2-M control is considered to be an important parameter for the evaluation of patients diagnosed with CRF or who are on hemodialysis. The normal level of BUN is 8-18 mg/dl, of serum Cr (11) it is 0.6-1.2 mg/dl, and of urea in saliva (12) it is 1.66-7.5 Mmol/l.

CRF can give rise to a wide spectrum of oral manifestations, including increased accumulation of plaque, gingivitis, bleeding gums, ammonia breath adore, candidiasis, glossitis, loss of trabeculation, loss of the laminadura, loose teeth, jaw bone demineralization, nonspecific lesions, and delayed tooth eruption. Hemodialysis treatment can decrease the severity of oral manifestations caused by renal failure. Tomas et al. (13) have conducted a study on changes in the salivary composition of patients with renal failure. The results of the study revealed that salivary composition in patients with CRF was conditioned by the stage of renal failure. World wide, the number of patients affected by oral manifestations will increase due to the increased prevalence of renal failure (4, 14).There have been several reports on the changes of salivary flow in patients with end stage renal disease (ESRD), but oral and dental aspects of renal disease are not yet clear and more investigations are required (4, 15, 16). The present study attempts to understand the relationship between salivary β2-M and intensity of uremia in patients with chronic renal disease.

## Materials and Methods

There were 42 males enrolled in this case-control study of which 21 were diagnosed with CRF and 21 were assigned to a control group. The re search was conducted in the Nephrology Department of Shahid Beheshti Teaching Hospital in Hamedan, Iran. CRF pre-dialysis patients glomerular filtration rate (GFR between 15-90 ml/min) had no other evidence of systemic disease. In addition, there were no drugs affecting the oral mucosa and saliva like medications for psychiatric disorders and blood pressure lowering drugs. The control group wasselected from among healthy volunteer donors who continuously referred to the Blood Transfusion Organization. Both groups were similar with respect to age, gender, and body weight. All were nonsmokers. Written informed consent was obtained from all study participants. Study was approved by Hamadan Medical Science University Ethics Committee.

All participants fasted overnight after which nonstimulated saliva samples were collected. The participants refrained from speaking during the saliva collection period. To prevent changes in salivary composition during the 24-hour period, participants were instructed not to eat, drink, or use a toothbrush, toothpaste, or mouthwash for 2 hours before sample collection. Saliva was collected by spitting into a test tube for 5 minutes after participants washed and rinsed their mouths. Saliva (10 cc) and venous samples (5 cc) were collected from research participants and delivered to the laboratory to determine serum and salivary β2-M, blood urea nitrogen (BUN), and Cr levels. The MININEPH HUMAN Kit with an autoanalyzer (Binding Site Co., UK) was used to measure β2-M levels by the nephrometric method. In this study, the amount of urea and Cr in saliva and blood samples was measured using the Pars Azmun Kit (Iran).

To measure serum β2-M, samples were diluted 1:40 and then placed in a spectrometer or autoanalyzer for 180 seconds. Obtained results were interpreted with the instrument. To measure salivary β2-M protein, saliva samples were diluted 1.5 times after which the remainder of the process was performed in the same manner as for the measurement of serum β2-M levels. GFR was calculated as follows:

$$\frac{\mathrm{(140-Patient’s Age)}\times \mathrm{Weight}}{72\times \mathrm{Serum Cr Level}}$$

Patients were divided into four groups: i. GRF ≥ 90 ml/min , ii. GFR between 60-90 ml/min, iii. GFR between 30-60 ml/min and iv. GFR between 15-30 ml/min. Pearson's correlation coefficient was used to calculate the correlation between salivary and serum markers. Independent t test compared the mean of each variable. Data were imported to SPSS version 15.

## Results

The mean age of patients diagnosed with CRF was 33.81 ± 6.45 years and of healthy people it was 49.48 ± 19.8 years. The mean weight of patients with CRF was 83.09 ± 11.2 kg and of controls, it was 67.29 ± 12.56 a similar age and weight range in both groups. The highest cause of renal failure was diabetes in 12 (57.7%) cases, followed by high blood pressure (28.6%), chronic glomerulonephritis (9.6%), and systemic lupus erythematosus (4.8%).

Table 1 shows the means in both healthy controls and patients of serum and saliva β2-M, urea, and Cr. Table 2 shows the comparison of these two markers in blood and saliva in both groups. There was a correlation between serum BUN level and salivary urea in both controls and patients which was statistically significant in the control group (p=0.028). The correlation between serum and salivary Cr was 0.195 (p=0.398) in controls and 0.598 (p=0.006) in CRF patients, which was statistically significant for patients. The relationship between serum β2-M and salivary levels was 0.133 (p=0.566) in the control group and 0.078 (p=0.737) for CRF patients, which was not statistically significant. The correlation between serum BUN and β2-M was 0.168 (p=0.469) in controls and 0.629 (p=0.002) in patients, which was statistically significant inpatients. The correlation between serum Cr and β2-M was 0.110 (p=0.635) in the control group and 0.678 (p=0.001) for CRF patients, which was statistically significant. The correlation between serum BUN and salivary β2-M levels was 0.093 (p=0.0690) in controls and 0.152 (p=0.152) for patients, which was not statistically significant. The correlation between serum Cr and salivary β2-M was 0.072 (p=0.070) in controls and 0.286 (p=0.209) in patients, which was not statistically significant.

**Table 1:** Average serum and salivary β2-M, urea, and Cr levels in chronic renal failure patients and control (healthy) subjects


Group	Diagnostic marker	Average	r	P value

Controls	Salivary Cr	0.8381 ± 0.22	0.195	0.398
	Serum Cr	1.00 ± 0.921		
	Salivary urea	20.333 ± 4.74	0.048	0.028
	Serum urea	21.667 ± 3.98		
	Salivary β2-M	0.9226 ± 0.52	0.133	0.566
	Serum β2-M	1.7662 ± 0.41		
Patients	Salivary Cr	0.759 ± 0.83	0.579	0.006
	Serum Cr	2.7381 ± 7.1		
	Salivary urea	44.05 ± 67.21	0.0401	0.072
	Serum urea	49.095 ± 36.21		
	Salivary β2-M	1.5857 ± 0.12	0.078	0.737
	Serum β2-M	5.9 ± 0.17		


r; Pearson Correlation Coefficient.

**Table 2:** Comparison of serum and saliva diagnostic markers between controls and patients


β2-M serum Serum BUN	Healthy	0.168	0.469
Serum β2-M BUNSerum	Patient	0.692	0.002
Serum β2-M Serum Cr	Healthy	0.110	0.635
Serum β2-M Serum Cr	Patient	0.678	0.001
Salivary β2-M BUN Serum	Healthy	0.093	0.690
Salivary β2-M BUN Serum	Patient	0.152	0.510
Serum β2-M Serum Cr	Healthy	0.072	0.570
Serum β2-M Serum Cr	Patient	0.286	0.209

r; Pearson Correlation Coefficient.

## 

In the present study there was no significant difference between salivary β2-M and urea levels serum, Cr and BUN) in CRF patients. In this study, 12 out of 21 CRF patients were diagnosed with diabetes. These results were consistent with those reported by Proctor et al. (3), Erturul et al. (4), and Afshar et al. (17). The median age in healthy subjects was 49.4 years and in CRF was 33.8 years. The median age in patients with chronic renal disease in a study conducted by Afshar et al. (17) was 51.6 ± 17 years, which was consistent with the current study. The average value of salivary urea concentration was 44.048 Mmol/l in patients, which approximated the results reported by Haag et al. (14). Normal salivary urea levels are within the range of 1.66-7.5 Mmol/l in healthy human subjects as defined by Chung et al. (18). In our study the mean salivary urea was found to be 20.33 among healthy human subjects and was higher than reported by Chung et al. (24). No significant difference was found between the present and a previous study conducted by Borhan Mojabi et al. (19) regarding mean salivary urea (20.33 vs. 14.85 Mmol/l) levels. According to Tomas et al. (25), the mean salivary urea in healthy human subjects was 7.56 Mmol/l and, in patients with progressive chronic kidney disease it was 17.03 Mmol/land in patients with non-progressive chronic kidney disease it was 26.28 Mmol/l.

In the current study, the mean Cr concentration in saliva of healthy human subjects was measured at 0.838 µmol/l, while it was reported as 0.3250 µmol/l in patients with stage I CRF, 0.4333 µmol/l (stage II CRF), 0.5875 µmol/l (stage III CRF), and 1.1625 µmol/l (stage IV CRF). In a study carried out by Tomas et al. (13) in 2008, the salivary Cr level in healthy human subjects was 0.08 µmol/l, in patients with progressive CRF it was 0.211 µmol/l and in non-progressive CRF patients the level was 0.784 µmol/l.

Vesterinen et al. (20) in 2010 found that high levels of urea and Cr in CRF patients led to poor oral health. In the present study, the median level of salivary β2-M in healthy human subjects was 0.9226 mg/l, and in patients it was 1.25 mg/l (stage I CRF), 0.9333 mg/l (stage II CRF), 2.1875 mg/l (stage III CRF), and 2.3125 mg/l (stage IV CRF). The median serum β2-M level in controls was 1 mg/l, and inpatients it was 3.4 mg/l (stage I CRF), 3.2333 mg/l (stage II CRF), 6.4875 mg/l (stage III CRF), and 6.6625 mg/l (stage IV CRF). In a study conducted by Michelis et al. (21) the median level of salivary β2-M in healthy human subjects was 0.9 mg/l and in patients with advanced renal failure it was 1.0 mg/l. The results of the present trial concerning salivary β2-M agreed with the Michelis et al. (21) study. The results of our study were compatible with results reported by Akalin et al. (22) who studied β2-M levels in serum and saliva of patients with juvenile periodontitis (JP) and healthy human subjects. Their results showed that the level was higher in serum of patients with JP compared to healthy subjects, but showed no difference in saliva levels. This marker possibly plays a role as a systemic factor in the development of this disease. In another study conducted by Crisp et al. (9), the researchers found that salivary β2-M in healthy human subjects was between 0-0.38 mg/l. Significant differences between our study and the Crisp et al. study might be the result of differences in laboratory techniques.

In the present study, we used the MININEPH HUMAN Kit and Pars Azmun Kit instead of an immunoassay kit for nephelometric measurements. Crisp et al. (9) reported a median serum β2-M level in healthy human subjects of 1.2 mg/l and in patients with advanced renal failure the level was 9.5 mg/l. In another study conducted by Regina et al. (23) in 2007, they found that patients who developed CRF had higher serum β2-M levels, and these levels were elevated in patients on hemodialysis.

In a study conducted by Mehal et al. (24) the predialysis serum β2-M level was 46.7 ± 3.9 mg/l. However the duration of dialysis had no effect on β2-M levels. The reason for this marked difference in serum β2-M levels between our study and the previous one carried out by Mehal et al. (24)was because we did not include hemodialysis patients in our study. In a study conducted by Sapvander et al. (25) the β2-M concentrations were measured by radioimmunoassay. The median serum BUN in healthy human subjects was 21.667 mg/dl, in patients with CRF it was 22 mg/dl (stage I), 29.667 mg/dl (stage II), 42 mg/dl (stage III), and 70.25 mg/dl (stage IV). Tomas et al. (13) found that patients with renal failure were more likely to have elevated serum BUN levels. According to the results of the previous study, the serum BUN level in healthy human subjects was 5.51 ± 1.04 mg/ dl; in patients with advanced and early stages of renal failure it was 18.80 ± 17.88 mg/dl for advanced renal failure and 26.86 ± 8.43 mg/dl for early renal failure, respectively. There was a strong correlation between serum BUN, severity of kidney failure, and salivary composition amongthe studied participants.

Salivary urea level and its relation to oral and dental health have been studied by Vesterinen et al. (20). The results of their study reveal that urinary urea levels in patients with advanced renal failure are in agreement with mean values of those in the present study in patients diagnosed with stages I and II CRF. In a study by Bayraktar et al. (26) the mean serum Cr in the control group was 1.00048 mg/dl and in CRF patients it was 1.6 mg/dl (stage I), 1.4 mg/dl (stage II), 2.037 mg/dl (stage III), and 4.225 mg/dl (stage IV). Serum Cr levels in patients with CRF and acute renal failure treated with peritoneal dialysis and hemodialysis was 55 and 142 mg/dl respectively which was a significant difference with the control group. By excluding dialysis patients, there was a significant difference between the results of our study and those published by Bayraktar et al. (26). In a study by Tomas et al. (17) the Cr level in the control group was 80.53 ± 13.54 µmol/ land in patients with advanced and early renal failure the levels were 211.32 ± 221.00 and 784 ± 213.35 µmol/l, respectively which agreed with our study. Showing rise in Cr levels in patients with advanced renal failure compared to control group.

## Conclusion

There wasno significant relationship between salivary β2-M and serum urea and Cr levels in this study, therefore salivary β2-M cannot be used as a non-invasive indicator to detect the intensity of uremia in patients with chronic renal disease. According to the results of this study, it seems logical to detect uremia intensity using serum β2-M. We suggest that since the salivary β2-M concentration is affected by a number of factors, including oral hygiene and drug therapy, further studies are strongly recommended. We also recommend to examine salivary β2-M concentration in ESRD patients receiving hemodialysis.
